# Syntax at Hand: Common Syntactic Structures for Actions and Language

**DOI:** 10.1371/journal.pone.0072677

**Published:** 2013-08-22

**Authors:** Alice C. Roy, Aurore Curie, Tatjana Nazir, Yves Paulignan, Vincent des Portes, Pierre Fourneret, Viviane Deprez

**Affiliations:** 1 L2C2- Institut des Sciences Cognitives, CNRS UMR 5304, Bron, France; 2 Université Claude Bernard Lyon I, Lyon, France; 3 Centre de Référence «Déficiences Intellectuelles de Causes Rares» Hôpital Femme Mère Enfant, Hospices Civils de Lyon, Bron, France; 4 Service de Psychopathologie du Développement- Hôpital Femme Mère Enfant, Hospices Civils de Lyon, Bron, France; 5 Université de Lyon, Faculté de Médecine Lyon Sud - Charles Mérieux, Lyon, France; University of Milan, Italy

## Abstract

Evidence that the motor and the linguistic systems share common syntactic representations would open new perspectives on language evolution. Here, crossing disciplinary boundaries, we explore potential parallels between the structure of simple actions and that of sentences. First, examining Typically Developing (TD) children displacing a bottle with or without knowledge of its weight prior to movement onset, we provide kinematic evidence that the sub-phases of this displacing action (reaching + moving the bottle) manifest a structure akin to linguistic embedded dependencies. Then, using the same motor task, we reveal that children suffering from specific language impairment (SLI), whose core deficit affects syntactic embedding and dependencies, manifest specific structural motor anomalies parallel to their linguistic deficits. In contrast to TD children, SLI children performed the displacing-action as if its sub-phases were juxtaposed rather than embedded. The specificity of SLI’s structural motor deficit was confirmed by testing an additional control group: Fragile-X Syndrome patients, whose language capacity, though delayed, comparatively spares embedded dependencies, displayed slower but structurally normal motor performances. By identifying the presence of structural representations and dependency computations in the motor system and by showing their selective deficit in SLI patients, these findings point to a potential motor origin for language syntax.

## Introduction

The nature of the relationships between language and motor control is currently the object of growing attention [1]. Up to now, however, empirical research has largely centered on the meaning of action words and their representation in our sensory and motor systems [2 for a review]. With their focus on the lexico-semantic domain, these studies have rarely addressed other core aspects of language such as its structural aspects and its syntax. Yet, the common involvement of Broca’s area in syntax and in the sensori-motor system points to possible convergence between these domains [3,4]. Positive evidence that syntax-based representations could be partially common to the motor and the linguistic systems would suggest that linguistic syntax could have exploited and built upon parts of a pre-existing “syntax” used by the motor system [5–7]. The present study provides support for this assumption.

Like verbs in spoken language, actions arguably manifest a comparable argument structure relating agents and objects [8]. Accordingly, they are commonly branded as ‘transitive’ when performed with or towards objects [9]. Knott [10] for instance, proposes that "the Logical Form" of a sentence reporting a cup-grabbing episode can be understood as a description of the sensorimotor processes involved in experiencing the episode. He argues that the LF of the sentence can be given a detailed sensorimotor characterization, and that many of the syntactic principles are actually sensorimotor in origin.

Similarly, drawing on modeling studies of motor planning, Jackendoff [11,12] suggested that actions are recursively structured in ways quite analogous to the hierarchical embedding that characterizes language syntax and further conjectured that the structure of certain sub-events within goal-oriented actions (e.g. preparing coffee) could even have "the flavor of variable binding and long distance dependencies" [12 p597] like those at play in the syntactic structures of questions or relative clauses. Jackendoff [12 p597] describes one such dependencies in the complex routine action of making coffee as follows: "For instance, suppose you go to take the coffee out of the freezer and discover you’re out of coffee. Then you may append to the [making coffee] structure a giant preparation of buying coffee, which involves finding your car keys, driving to the store, going into the store (…) and driving home. The crucial thing is that in this deeply embedded head (i.e. the buying action), what you take off the shelf (...is...) the same thing you need in the larger structure this action is embedded in".

**Figure 1:**
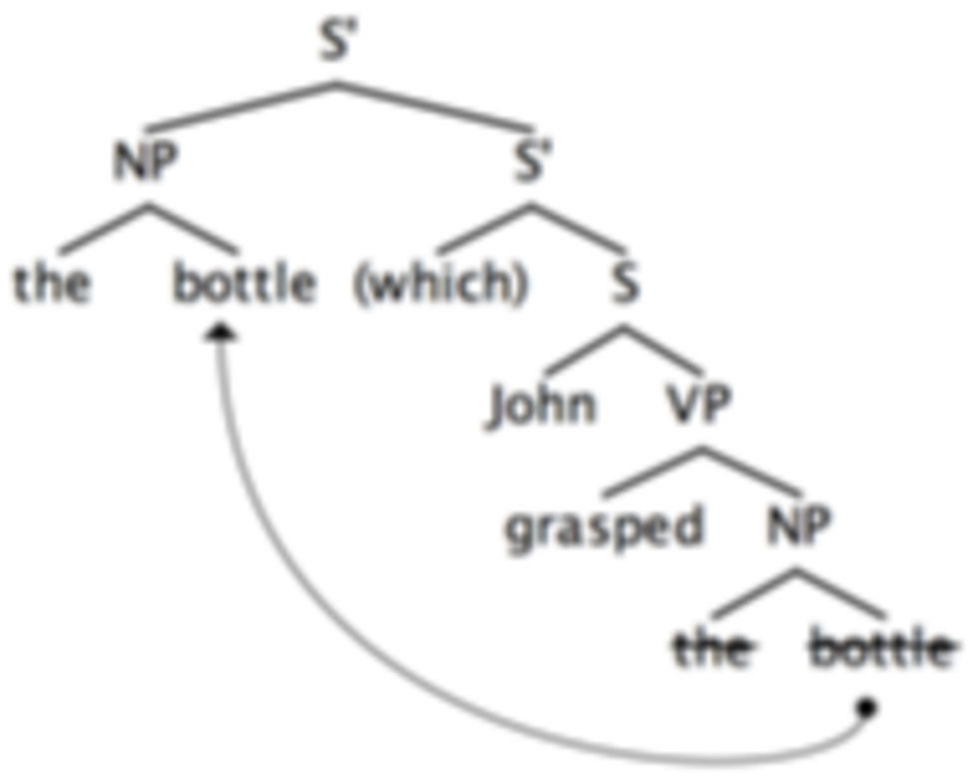
Representation of the syntactic structure of an embedded clause. The noun phrase "the bottle" appears twice, once when it is pronounced at the beginning of the main clause and as a trace in the position it is interpreted in i.e. as the object complement of the verb grasp of the relative clause. Note that this structure is characteristically asymmetric.

Consider now in some detail the syntactic structure of a relative clause dependency in language. In a simple sentence like [ *Johngrasped* *the bottle*], the complement of a transitive verb like “*grasped*”, i.e the nominal phrase [*the bottle*], generally occurs after the verb. In contrast, in a relative clause like ‘ [*This is the bottle (which)* *Johngrasped* ] the nominal phrase [*the bottle*] is syntactically displaced to the front of the clause, so that it no longer appears after the verb. Yet, despite this displacement, the nominal phrase remains interpreted as the complement of the verb “*grasped*”. In syntactic theory, the link between the displaced position of a fronted nominal phrase and the position in which it is interpreted (i.e. here as a complement of the verb grasp) is modeled as a ‘distant dependency’ between the two syntactic positions of the nominal phrase. Syntacticians propose that a relative clause is a transform of a basic sentence in which the nominal complement of a verb has been displaced to the front of a clause leaving a silent copy in the position in which it originated and is interpreted e.g. [*This is the bottle (which)* *Johngrasped* *the bottle*]. Schematically, the abstract structure of a relative clause is as in Figure 1: Here, S’ represents the relative clause, S, the original sentence, the arrow indicates the displacement i.e. the distant dependency between the nominal phrase (NP) and its silent copy represented here as the crossed NP : *the bottle*.

‘Distant dependencies’ are also known in linguistics as filler-gap dependencies or operator-variable dependencies. All these dependencies involve relations akin to that between a noun and a pronoun that is two representations of the same element as in: (John thinks he will win, where ‘he’ = john) but where the pronoun is silent. ‘Distant dependencies’ have played a central role in linguistic theory in probing the hierarchical nature of sentence embedding [13]. As Ross [14] showed indeed, these dependencies are tightly constrained. In particular, although the distance between the displaced nominal phrase and its silent copy can span over several clauses if these are embedded, the dependency fails to be properly interpreted if the intervening clauses are coordinated or juxtaposed instead. Compare the following examples:

(a). This is the heavy bottle (which) [John realized] (that) [Mary knows] (that) [he grasped [the bottle]]

(b). * This is the heavy bottle (which) [John smiled] (and) [Mary stretched] and [he grasped [the bottle]]

**Figure 2:**
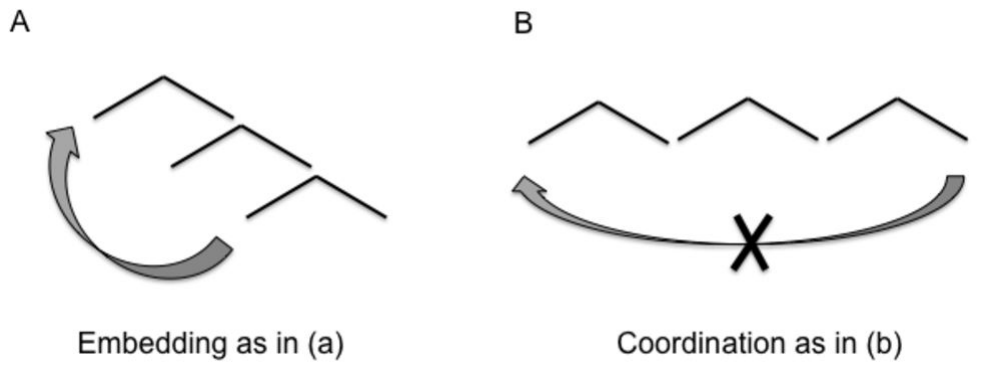
Schema for embedded and coordinated sentence structure. A: An embedded structure is essentially asymmetric and accepts distance dependency as in the example given in (a). By contrast a symmetric coordinated structure does not accept distance dependency.

Please note that the asterisk in front of the sentence indicates that the sentence in (b) is ungrammatical and that the elements in parenthesis are optional in English. They can remain unpronounced so that respectively the two sentences can be realized as:

This is the heavy bottle John realized Mary knows he grasped vs. This is the heavy bottle John smiled Mary stretched and he grasped. In (a), the dependency between the fronted nominal phrase, *the bottle* and the position in which it is interpreted (e.g. as a complement of the verb grasp) spans over three embedded clauses, but the sentence is still natural. In (b) -though formed from the perfectly accep It should be noted that we are not claiming that motor syntax is as complex as linguistic syntax, nor that it shares all of its crucial aspects, but we are arguing that some rudimentary, but fundamental aspect of linguistic syntax could be traced back to relatively complex motor actions. sentence "John smiled and Mary stretched and he grasped the heavy bottle"- it is rather difficult to understand the fronted nominal phrase [*the bottle*] as the complement of the verb grasp and still obtain a fully natural sentence. Note that the actual linear distance between the fronted noun phrase and the silent copy [*the bottle*] after the verb grasp is the same in (a) and (b). What differs is the nature of the syntactic relationship that the intervening clauses entertain: embedded vs. coordinated. That is, what matters is the nature of the abstract structural relation that connects the components that intervene between the two pieces of the distant dependency, the nominal phrase and its silent copy. Hence, the break down of the distance dependency in (b) vs. (a) serves to reveal a fundamental distinction in the syntax of these two sentences that would otherwise not be immediately obvious from the simple sequential ordering of their components. The distinction in the structural relations between the component constituents is schematized in Figure 2.

As the arrows indicate, a linguistic distant dependency can succeed when the intervening components are embedded, but it breaks down when they are coordinated or juxtaposed. This structural constraint, dubbed the "Coordinated Structure Constraint" in the linguistic literature, provides evidence of the fundamentally hierarchical and embedded nature of certain linguistic structures.

Following Jackendoff’s conjecture that comparable distant dependencies are found in the motor domain, we sought to use these to experimentally probe the nature of the abstract structural relations that connect the motor components of a transitive action.

Endeavoring to construct an analogous motor distant dependency, we used a basic goal-directed displacing-action task that consisted in reaching a bottle and moving it to another target location while manipulating the participant’s knowledge of its weight. Our task was designed, first, to build on existing evidence that a displacing motor action can be divided in two component sub-phases [15], a first sub-phase of [reach+grip] that unfolds before object contact, dubbed here the Reach sub-phase, and a second sub-phase of [lift+move] that unfolds after object contact, dubbed here the Move sub-phase (Figure S1). Second, we chose to manipulate the weight of an opaque bottle because, in contrast to features such as shape, size or location, which are visually perceived and hence can come to affect the kinematics of a reach and grasp action before any object contact [16], weight is a somatosensory perception that affects movement kinematics only after object contact [17–19] as long as there are no visual (or other) cues to it. That is, weight information, is normally "encapsulated" in the second phase of a displace action, i.e. dubbed here the Move sub-phase. Yet, weight information could become accessible prior to object contact [e.g. 20-22], if the weight of a given object is experienced or known before critical movement onset. When known, weight information could be so to speak ‘raised out’ of the Move sub-phase where it is normally encapsulated, and become accessible to potentially affect kinematic parameters already in the Reach sub-phase. Such a displaced weight effect in the Reach sub-phase would form the motor equivalent of a ‘distant dependency’ between the point in the Reach sub-phase where the mental representation of object weight is integrated in movement kinematics and the point of object contact in the Move sub-phase where the object weight is physically accessed. Note that at this second point in the action structure i.e. at the point of contact, the pertinent object weight better matches the representation integrated earlier in the Reach component, or else a mismatch would occur. This implies that a representation of object weight must be part of the motor computation to allow a backward feedback similar to the relation posited in linguistics between a displaced nominal phrase and its silent copy.

**Figure 3:**
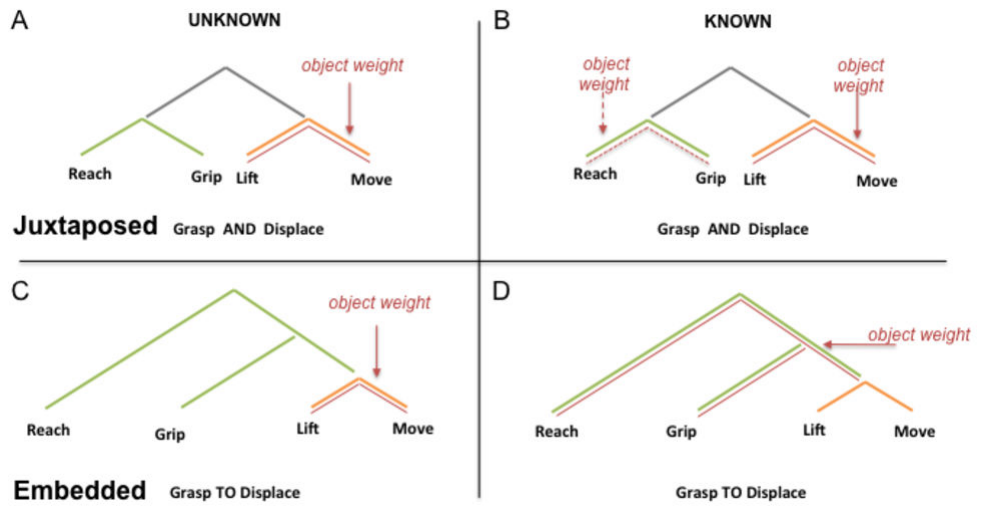
Potential structures of the two sub-phases of a displacing action. AB: Sequential juxtaposition, analogous to [I reach-grip a bottle AND lift-move it]. When object weight is unknown prior to object contact (A), kinematic parameters adapt to object weight in the ‘Move sub-phase’ (schematically indicated by the red line). No effect of object weight is expected in the ‘Reach sub-phase’. By contrast, when object weight is known in advance (B), movement kinematics could differ for heavy and light objects conjointly in the ‘Reach sub-phase’ and the Move sub-phase i.e. [This heavy bottle, I reached-griped it AND lift-moved it]. CD: Syntactic embedding, analogous to [I reach-grip a bottle TO lift-move it]. When object weight is unknown kinematic parameters adapt to object weight in the ‘Move sub-phase’ only (C). When known prior to movement onset object weight effects could be front-moved from the Move sub-phase to the reach sub-phase(D), following this displacement the Move sub-phase kinematics would remain immune to object weight effects (i.e., [The heavy bottle that I reached-griped TO lift-move]).

Although there is a rather broad consensus in the motor literature that a simple object displacing action of the type we used can be subdivided into two distinctive sub-components [15], the nature of the structural relation between these two components has at this point not been investigated empirically. A priori, the two sub-phases of a displacing action could be structurally related in either of two ways: they could be merely sequentially juxtaposed in analogy with the coordinated linguistic structures in Figures 2 and 3A-B or entertain a complex hierarchical relation, a form of syntactic embedding, analogous to the embedded structure in Figures 2 and 3C-D. This subtle but key distinction is essential for comparing motor with linguistic structure because in language, as illustrated in Figure 2, the two types of structural relations have characteristically distinct effects on distant dependencies. While juxtaposed constituents are largely independent from one another, i.e. there have no domination or inclusion relation, embedded constituents are hierarchically related and thus asymmetrically dependent with domination or inclusion relations. Manipulating access to object weight information, during or prior to action execution, by creating a distant dependency, allows us to probe the structural relation entertained by the two component sub-phases of a displacing action. When the weight of an object is unknown to the subject, the kinematic parameters should always adjust to weight after object contact, that is, in the Move sub-phase only: such cases, then do not afford the possibility to uncover a distinction between the two potential structural models for our displacing action depicted in Figure 3. However, when the object-weight is known in advance, so that a potential distant dependency now arises between a motor weight representation before object contact and the point of object contact where weight is physically felt, the two models make different predictions. If the displacing-action has a juxtaposed structure (with two relatively independent and parallel sub-components), weight effects could be distributed over and affect the kinematic parameters of both sub-components symmetrically (red line in Figure 3) in analogy with what happens in a linguistic coordinated structure as the following (e.g. the heavy/light bottle, I reach it AND I move it) where the fronted nominal complement ‘[the heavy/light bottle] is resumed by an overt pronoun (it) in both constituents of the coordinated structure. That is, if the structure of the displacing action is as in Figure 3B, we expect weight to affect both the Reach sub-phase as the weight representation is accessible to affect the kinematic computation, and the Move sub-phase as this is where the object weight is actually felt. By contrast, if the two components of the displacing action are embedded and object properties computation is akin to a linguistic distant dependency, as we conjecture, prior weight knowledge could result in an asymmetric transfer of the weight effects to the topmost level of the hierarchical structure, i.e., to the ‘Reach sub-phase’ with a consequent reduction or absence of weight effects in the ‘Move sub-phase’ in analogy with the silent copy that a linguistic dependency leaves in the original complement position when a nominal phrase is displaced (e.g. the heavy/light bottle which I reach to move [the bottle]). In similarity with linguistic displacement, object weight effects could be so to speak ‘raised out’ of the Move sub-phase and be displaced to affect the Reach sub-phase, leaving in turn the kinematics parameters of the lower Move sub-component unaffected by weight, in analogy with the linguistic silent copy left after fronting in relative clauses [23].

To test this hypothesis and probe the structural relation of the motor components of a simple structured action, we compared the behavior of Typically Developing (TD) children with that of children diagnosed with Specific Language Impairment (SLI). SLI refers to a deficit in language acquisition that occurs in children who are otherwise developing normal cognitive abilities (absence of mental retardation, no diagnosed motor deficit, no hearing loss, or identifiable neurological disease). We reasoned that if the two sub-components of our displacing action presented a hierarchical structure rather than a mere juxtaposition, SLI patients whose core deficit has been shown to affect among other complex hierarchical sentences and, even more specifically, relative clause structures [24–27] may present a parallel deficit in performing a structured motor action. Such evidence would support the hypothesis that motor control and syntax could share analog mechanisms, possibly grounded in common neural structures. Both groups of children were asked to displace one of two identically looking opaque bottles with a significant weight difference (50g vs. 500g). The bottle was placed at a fixed distance from the participants’ hand, under two weight-knowledge conditions: one in which the weight was unknown prior to movement execution (Unknown condition) and one in which weight was known in advance (Known condition). Participants were first familiarized with the two object weights to ensure they had acquired sensorimotor knowledge of each before kinematic acquisition. In the unknown condition, the bottles were presented with a random alternation in weight unbeknownst to the participants, so that they did not get information about the object weight until object contact in the Move sub-phase. In the known condition, in contrast, participants were provided with the relevant information about the object weight before movement execution, and trials with a specified weight were presented consecutively. TD’s behavior enabled us to uncover the structural representation of the displacing-action, while the behavior of the language impaired population allowed us to trace potential parallels between motor and linguistic impairment.

## Results

The distinction between the two structures depicted in Figure 3 rests on modulations of the effect of physical object weight as a function of prior weight-knowledge. Our analysis therefore focused on interactions between the factors Weight and Knowledge in each of the two sub-phases of the displacing-action. Whenever this interaction was significant, planned comparisons between heavy and light objects were performed. Additional statistics are given in Table S1 in File S1.

**Figure 4:**
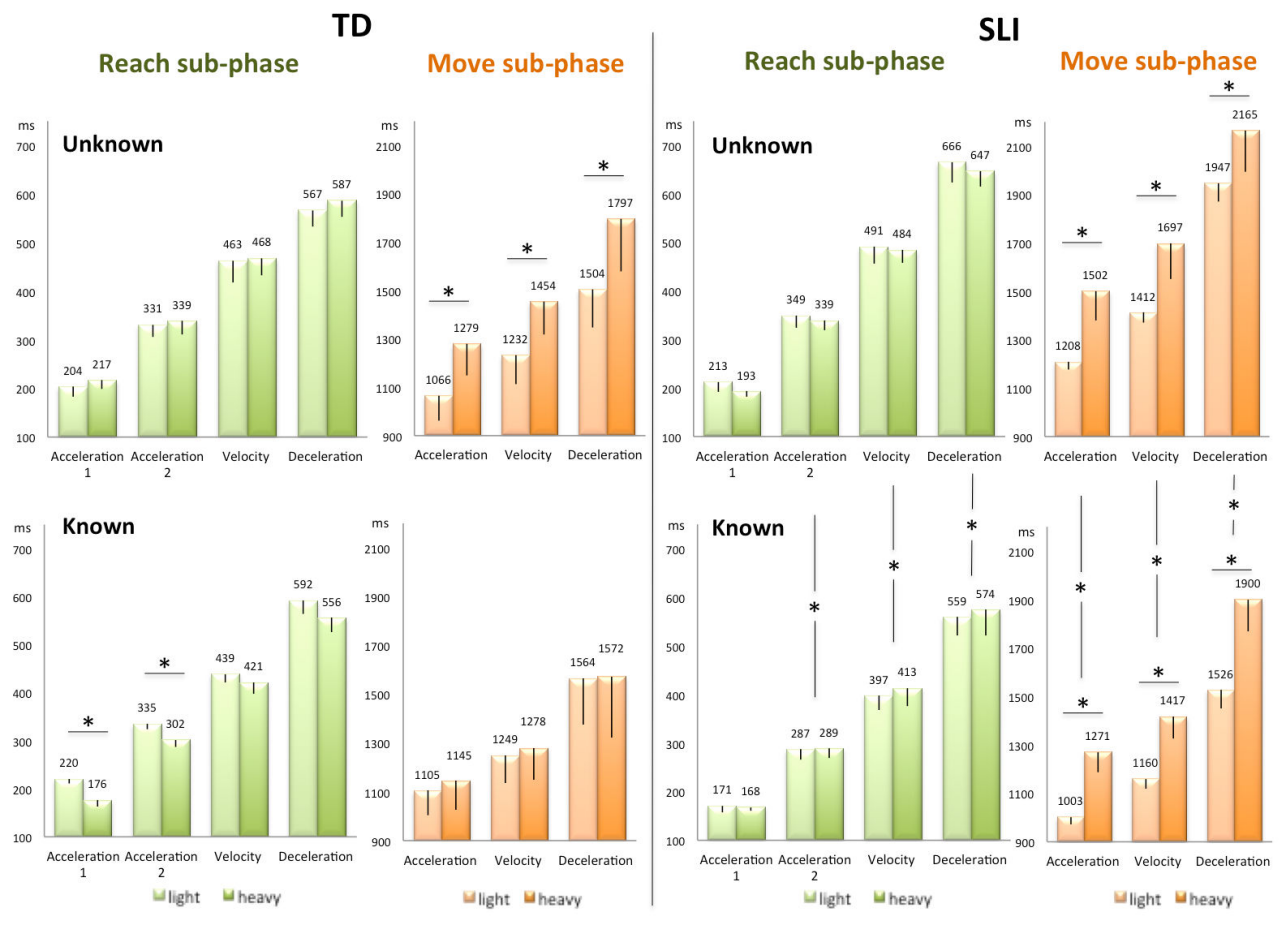
Kinematic parameters latencies in the Reach sub-phase (green) and the Move sub-phase (orange) for TD (left) and SLI children (right) for the unknown (top) and known (bottom) conditions. Horizontal line with (*) indicates significant planned comparison between heavy and light objects. Vertical line with (*) indicates significant main effects of Knowledge (all p<= .05).

**TD children** (n=7, 4 males, mean age 10 years and 6 months). Figure 4 in the left panel plots peak latencies for the analyzed kinematic parameters (Figure S1). The critical interaction between Weight x Knowledge was observed for 4 parameters in the Reach- and 3 parameters in the Move sub-phases. Additionally the interaction between Weight x Knowledge was found on the whole action time (the time elapsed from the beginning of the Reach sub-phase to the very end of the Move sub-phase when the hand left the bottle). Planned comparison between heavy and light objects in the Known and Unknown conditions revealed that:

in the **Unknown condition**, when the weight was unknown prior to object contact, its effects were absent from the Reach sub-phase, and present only in the Move sub-phase. In this Move sub-phase, we found that heavy objects gave rise to delayed wrist acceleration peaks (F^1,6^=27.09; p=.002), velocity peaks (F^1,6^=27.64; p=.001), and deceleration peaks (F^1,6^=11.40; p=.01) as compared to light objects (Figure 4). The amplitude of the acceleration peak and of the velocity peak were also smaller when displacing the heavy compared to the light objects, however, the interaction between Weight x Knowledge remained marginally significant (Table S1 in File S1). In sum, displacing the heavy object as opposed to the light one had a cost (delayed and decreased peaks) that translated in an overall longer whole action time (F^1,6^=6.68; p=.041).

In contrast, in the **Known condition**, when the object-weight was known in advance, the kinematic parameters adapted to the weight already in the Reach sub-phase. Reaching for the heavy objects yielded anticipated wrist acceleration peaks and maximum grip aperture (latency of first acceleration peak F^1,6^=12.24; p=.01; latency of second acceleration peak F^1,6^=7.89; p=.03; latency of maximum grip aperture F^1,6^=23.72; p=.003). Even more noticeably, weight effects failed to be evident in the Move sub-phase (Figure 4); the light and heavy objects gave rise to equivalent profiles for the relevant kinematic parameters, even though direct contact with the object occurred only in this second sub-phase. Characteristically, in the Known condition, TD children exhibited a comparable whole action time for the two object weights, as if the cost of displacing a heavier object had been counterbalanced during the Reach sub-phase by an anticipation of its kinematics consequences. Note that the object weight effects in the Move sub-phase of the Unknown condition were the inverse of the object weight effects observed in the Reach sub-phase of the Known condition. That is, heavy objects gave rise to later and smaller peaks in the Move sub-phase of the Unknown condition, while expected heavy objects gave rise to earlier peaks in the Reach sub-phase of the Known condition. These anticipated peaks are readily understandable as a motor strategy to compensate the added cost of moving the heavy object on the whole action time, which was observed in the Unknown condition.

**SLI children** (n=7, 4 males, mean age 11 years, p = ns with respect to TD children age). None of the measured motor parameters (latencies or amplitudes) showed an interaction between Weight x Knowledge (Figure 4 right panel; Table S1 in File S1).

In the **Unknown and Known conditions**, object weight effects were strictly confined to the Move sub-phase (that is after direct contact with the bottle had occurred). Moving the heavy object as compared to the lighter one resulted in later and smaller peaks (main effect of Weight on latencies of acceleration F^1,6^=29.93; p=.002, velocity F^1,6^=31.60; p=.001, and deceleration peaks F^1,6^=14.73; p=.009; main effect of Weight on amplitudes of acceleration F^1,6^=14.48; p=.009 and velocity F^1,6^=16.84; p=.006). The cost of object weight in the Move sub-phases was such that it impacted the whole action time in both the Unknown and known conditions (main effect of Weight on whole action time F^1,6^=42.92; p=.001).

Additionally in the **Known condition**, a symmetric main effect of Knowledge was observed in both the Reach and the Move sub-phases, consisting in shorter latencies (for both light and heavy objects alike) when object weight was known in advance by SLI children (In the Reach sub-phase: Time to second acceleration F^1,6^=10.39; p=.018, velocity F^1,6^=10.88; p=.016 and deceleration peaks F^1,6^=9.81; p=.02; In the Move sub-phase: Time to acceleration F^1,6^=5.88; p=.051, velocity F^1,6^=10.38; p=.016, and deceleration peaks F^1,6^=13.17; p=.011). Finally, the whole action time was overall shorter in the Known condition than in the Unknown condition (Main effect of Knowledge on whole action time F^1,6^=8.37; p=.028). These kinematic effects (i.e shorter latencies and whole action time in the Known condition) crucially testify that SLI children did not simply ignore weight information. In the Known condition, they clearly integrated the weight information of the object, but for them this information affected both subcomponents symmetrically. That is, knowing *that* the object was heavy/light did not translate into knowing *how* to shift the weight effects to the Reach component to adapt kinematic parameters appropriately.

Importantly, our results for the Unknown condition also provide evidence that SLI children did not otherwise show any gross motor impairment nor specific problem in dealing with object weight; when the object weight was unknown, SLI performance did not differ from those of TD children. Accordingly, an omnibus non parametric MANOVA performed with Group (TD, SLI) as a between-subject factor and Weight (heavy, light) and Knowledge (known, unknown) as within-subject factors revealed no main effect of Group (F=11.87; p = ns), but a significant three way interaction (F=38.49; p=.018). A non parametric MANOVA performed for each group separately, further confirmed that the within-subject factors Weight and Knowledge interacted in TD children movements (F=45.73; p=.01) but not in SLI children movements (F=11.32; p = ns).

### Specificity of the syntactic motor deficit

To ascertain the specificity of SLI motor deficit we examined the performance of a group of Fragile X Syndrome (FXS) patients on the same motor task. FXS is the most common cause of inherited intellectual disabilities and the most common single gene cause of autism (90% of FXS patients present autistic-like behavior [28]). FXS offer an appropriate control for potential confounding factors coming from reduced cognitive resources or autistic traits as the border between SLI and autistic disorder is blurred [29]. Most importantly language acquisition in FXS is delayedm with first words appearing at 26,4 months instead of 11 for TD and 23 for SLI [30,31], however, the profile of FXS’ linguistic impairment differs from that of SLI subjects, as it centrally concerns speech rate, articulation, and pragmatic aspects of language. Crucially, the syntactic level of FXS subjects is thought to be delayed rather than deviant [30,32,33], and complex structures and distant dependencies have been observed to be spared [34]. We therefore asked FXS patients and age-matched healthy adults to perform the very same structured motor tasks.

**Figure 5:**
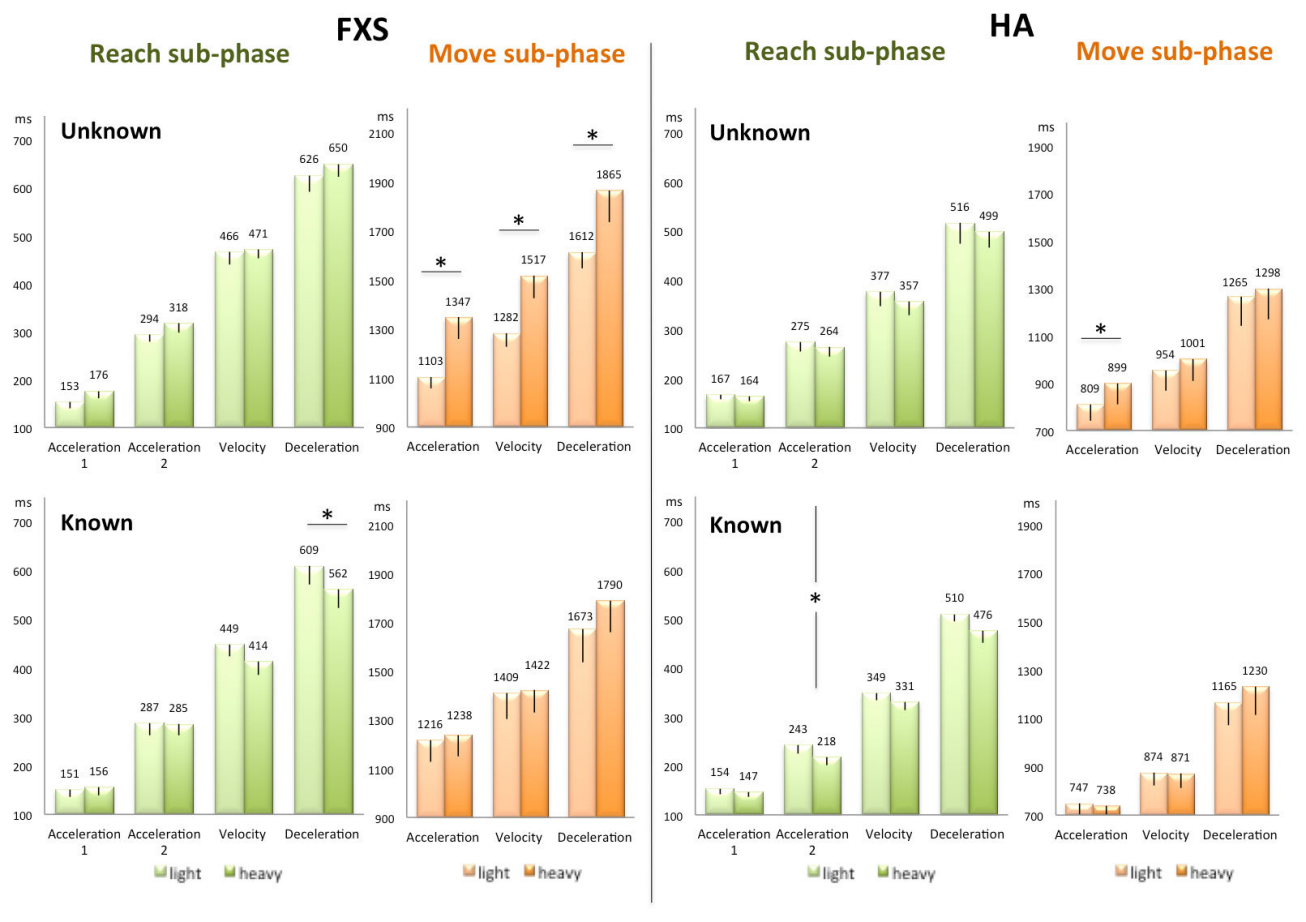
Kinematic parameters latencies in the Reach sub-phase and the Move sub-phase for FXS and HA. Same conventions as in Figure 4.

**FXS adults** (n=7, mean age 25 years). For FXS adults, like for TD children, the critical interaction between Weight x Knowledge was observed for peak latencies of several parameters in the Reach- and Move sub-phases and for the whole action time (Figure 5 left panel; Table S2 in File S1). Planned comparison between heavy and light objects in the Known and Unknown conditions revealed that:

in the **Unknown condition** when the object weight was unknown prior to contact, effects of weight were absent from the Reach sub-phase and present in the Move sub-phase only (latencies of acceleration F^1,6^=74.75; p<.001, velocity F^1,6^=37.95; p<.001, and deceleration peaks F^1,6^=28.73; p=.001) affecting nevertheless the whole action time (F^1,6^=14.21; p=.009). By contrast, in the **Known condition** the kinematic parameters adapted to weight in the Reach sub-phase (latency of deceleration peak F^1,6^=7.42; p=.03; latency of maximum grip aperture F^1,6^=17.5; p=.006) enabling the whole action time to be immune to the effects of object weight.

**Healthy Adults** (n=7, mean age 25,4 years, p = ns with respect to FXS age). . For healthy adults effects of object weight were generally small as further witnessed by the absence of weight effects on whole action time. Yet, like for FXS adults and TD children, the critical interaction between Weight x Knowledge was observed on peak latencies and amplitudes (Figure 5 right panel; Table S2 in File S1). Planned comparison between heavy and light objects in the Known and Unknown conditions revealed that:

in the **Unknown condition**, the effects of weight were absent from the Reach sub-phase and present in the Move sub-phase (planned comparison between heavy and light objects: acceleration peak latency F^1,6^=8.24; p=.02 and amplitude F^1,6^=18.54; p=.005). In contrast, in the **Known condition** when the object weight was known in advance, these effects were observed in the Reach sub-phase (planned comparison between heavy and light objects: deceleration peak latency F^1,6^=6.88; p=.039) but no longer in the Move sub-phase.

An Omnibus non parametric MANOVA with [Group (FXS, HA) as a between-subject factor and Weight (heavy, light) and Knowledge (known, unknown) as within-subject factors] revealed 1) a main effect of the factor Group (F=72.06; p=.004), FXS patients showing reduced amplitudes and delayed latencies with respect to healthy individuals (Figure 5, Table S2 in File S1); 2) an interaction between Group x Weight x Knowledge (F=33.40; p=.03). The non parametric MANOVA performed for each group separately confirmed that the within factors Weight and Knowledge interacted in FXS (F=44.44; p=.015), a similar result was found in healthy individuals though it did not reach significance (F=27.84; p=.078).

In sum, while movement kinematic parameters of SLI and TD children occurred in the same latency range and exhibited comparable amplitudes, SLI children differed from their control group in the ability to modulate kinematic parameters as a function of action structure. FXS patients, in contrast, differed from their control group with respect to their general movement amplitudes and latencies, but did not display deficits in their ability to modulate kinematic parameters as a function of action structure.

## Discussion

Crossing disciplinary boundaries, we explored whether the structure of simple actions could manifest a hierarchical embedding revealed by distant dependencies. We provided experimental evidence that the two sub-phases of a displacing action are asymmetrically structured in ways that owes more to embedding than to mere temporal juxtaposition. By manipulating when participants access object weight information, we devised a way to build a motor distant dependency in order to probe the nature of the structural relation that the two sub-phases of a displacing action entertain. When the weight of a displaced object is unknown, accessibility to weight information is governed by object contact. Consequently, weight can have an impact on kinematics only in the second component of the displace action, i.e. our Move sub-phase, and this, independently of how the two sub-phases are structured (Fig. 3AB). However, when weight information is available prior to object contact, so that participants are able to form a motor representation of the object weight prior to the onset of movement, only an embedded structure predicts that the impact of object weight on kinematics could asymmetrically transfer to the topmost action sub-phase, i.e. our Reach sub-phase, and leave the subordinate sub-phase almost unaffected. This transfer of the weight impact to the kinematic parameters of the Reach sub-phase, attests of a link between the two subcomponents that goes beyond mere juxtaposition.

As predicted, in the unknown weight condition, our results show that for all groups of participants alike, the object weight affected the kinematic parameters of the Move sub-phase, that is only after object contact: thus, the object weight information and the object weight kinematic impact were entirely encapsulated within the Move sub-phase. In contrast, when object weight was known in advance, our kinematic results crucially revealed that for TD children, HA and FXS patients, the kinematic impact of object weight shifted to the Reach sub-phase and was no longer encapsulated in the Move sub-phase. More strikingly and more significantly, this weight effect transfer was further accompanied by an almost complete disappearance of the weight effects from the Move sub-phase, despite the fact this sub-component is where direct contact with the objet and the weight somatosensory feedback takes place. It is thus as if the kinematic impact of the object weight had been entirely relocated from the Move sub-phase to the Reach sub-phase, leaving only a silent motor copy for feedback checking. We argue that only an embedding structure, as illustrated in Figure 3D, accurately models this observed pattern because, although an anticipated weight effect on the first sub-component could also be expected in a juxtaposed structure, this symmetric structure neither predicts nor explains the here observed weight effect disappearance from the second sub-component. Juxtaposed components are understood to be such, because they do not entertain relations beyond that of sequential, temporal or spatial, ordering. Given that the disappearance of kinematic weight effects from the Move sub-phase is a consequence of their anticipated impact on the Reach sub-phase, this weight effect transfer clearly suggests that the two components entertain relations that go beyond mere sequential ordering.

The relation between the point of object weight integration in the movement kinematic of the Reach sub-component and the point of object contact in the Move sub-component (where the weight is felt) presents a strong analogy to a syntactic operation of relative clause displacement in linguistic syntax. When known, the weight of an object is integrated into motor programming and execution prior to direct sensory contact with the object. Likewise, in a relative clause, a nominal phrase is pronounced, i.e. integrated in the speech computation before the position in which it is semantically interpreted. Furthermore, in motor embedding, the early integration of weight effects in the dominating Reach sub-component licenses a ‘silent’ motor copy of object weight in the Move sub-phase, quite analogous to the ‘silent’ copy of a displaced nominal phrase that linguistic theory posits after noun-phrase displacement [13]. That is, although the object weight is accessed via somatosensation after object contact, it no longer impacts the kinematic parameter at that point because weight effects were integrated earlier at a higher level of the action structure. Finally, recall that characteristically in language, ‘distant dependencies’ have been observed to lead acceptable sentences only across clauses that are embedded (This is the heavy bottle [John realized (which) Mary knows [he grasped]]), but not across clauses that are coordinated or juxtaposed (* ‘This is the heavy bottle (which) John smiled and Mary stretched and he grasped’). This linguistic hierarchical constraint dubbed “The Coordinate Structure Constraint” [14] seems here to be echoed in the motor system.

To further investigate whether the uncovered motor syntactic mechanisms at play in our displacing action displayed some fundamental aspects typical of linguistic syntax, we tested the movement structure of children with SLI. SLI children suffer from a disorder known to disrupt the development of a full-blown linguistic syntax. In particular, as it has been repeatedly observed, children with SLI commonly fail to produce and understand complex embedding and distant dependencies, particularly in relative clauses [24,25,35,36 but see 37 for an alternative statistical learning deficit hypothesis]. Furthermore, when prompted to do so, they have been observed to produce juxtaposition of matrix clauses instead. The sentence “I did it with my teacher, he’s called Doris” (the English rendering of a sentence produced by a French SLI patient, taken from 25) is a characteristic example of such failed attempts for the embedded relative clause: I did it with my teacher who is called Doris. In SLI, this deficient relative clause rendering has been taken to evidence a failure in the ability to construct appropriately embedded syntactic structures [24,25,35,36]. Though future studies are needed to establish intra-subjects correlation between motor and linguistic deficits, our findings on the distinctions between TD and SLI in the motor domain highlight an intriguingly striking parallel with the typical linguistic syntactic deficits SLI children have been observed to exhibit. With weight knowledge available prior to movement onset, SLI children were the only group that failed to transfer the motor computation of kinematic object weight adjustment from the Move to the Reach sub-phase. That is, despite prior weight knowledge, kinematic adjustments to weight for SLI children continued to take place only after object contact, i.e. still in the Move sub-phase. Thus, no displacement of weight effects was observed for this group. Yet, weight information was not ignored: As witnessed by the overall shortening of the latencies in the known condition both in the Reach and in the Move sub-phase alike, SLI patients benefited from weight knowledge. However, while for TD children, prior weight knowledge caused the appearance of an asymmetric shift in how weight effects impacted the two sub-components of our structured displacing action, for SLI children in contrast, advanced weight knowledge affected the two subcomponents symmetrically. This is as if the structure of the SLI children displacing sub-phases were juxtaposed, rather than embedded, provoking a distributed effect of advanced weight knowledge as if the expected normal execution of a motor distant dependency was disrupted. This, we suggest, echoes the Coordinate Structure Constraint at play in language syntax.

The rather striking analogy here observed for SLI patients between specific known language difficulties with relative clauses and hitherto unnoticed fine-grained structural motor abnormalities, highlights the possibility of common syntactic mechanisms in language and motor domain with renewed vigor.

Within the framework of the mirror system, an analogous motor impairment has been reported for autistic children by Cattaneo and colleagues ([38], see also 39). The study investigated the ability of autistic children to understand the motor intentions of others. In their study the authors compared the electro-myographic activity of the mouth opening muscle of TD with that of high functioning autistic children. These subjects were observing and executing two action chains; 1) reaching and bringing to the mouth a piece of food, 2) reaching and putting in a container a piece of paper. Characteristically, in the bringing to the mouth action, for both observation and execution, TD children exhibited an anticipatory activity of the mouth-opening muscle that started during the reaching sub-phase until the end of the movement. In contrast, autistic children failed to exhibit a comparable anticipatory muscle activity. Mouth muscle activation was confined to the bringing phase. Although our results also report an analogous failure in SLI children to kinematically anticipate weight effects, the putative resemblance between the two types of motor execution failure may only be superficial. In Cattaneo et al.’s study the task involved the anticipation of the goal of an action and the impact of knowing this goal on motor parameters. In contrast, our task investigated how knowing the weight of an object allows participants to transfer and invert the effects of object weight from the Move to the Reach sub-phase. This transfer is independent of the goal (i.e. the displacement of the object), which in our case remains the same. Hence, Cattaneo and colleagues observed a *delay* of the onset of the mouth opening muscles activity in autistic population, compared to their controls. In our case, however, the qualitative shift of the way motor parameters adapt to weight knowledge from the “Move-“ to the “Reach-“ phase, which characterizes the performance of our control groups, is never seen in the population of SLI children. Most importantly, FXS patients, who suffer from language deficits observed to spare syntactic embedding and distant dependencies [34], displayed a preserved ability to adjust their motor parameters as a function of action structure requirements. Despite a profound alteration of their motor performance, as witnessed by the overall delay in their movement parameters and the relative sluggishness of their motor performance, FXS patients produced what we could call a structurally correct motor distant dependency.

To date, only few studies have empirically probed the nature of motor syntax [see, for a review, 7]. Hoen and colleagues showed that agrammatic patients trained with non-linguistic sequences could improve their performance with relative clause comprehension [40]. In the motor domain, Fazio and coworkers [41] documented the inability of agrammatic brain-damaged patients to correctly reorder frames taken from a video-clip showing a human action, while their ability to reorder physical events was preserved. Using repetitive Transcranial Magnetic Stimulation (rTMS) in a paradigm similar to the one used by Fazio et colleagues, Clerget and colleagues [42] suggested that Broca’s area played a role in understanding complex transitive actions. In an Electro-Encephalogram study [43], healthy participants presented with videos containing expectancy violations of common real-world actions (i.e. an electric iron used in an ongoing bread cutting action) displayed two event related potentials (ERP), one negative peaking around 400ms and one positive peaking around 600ms and centered over the parietal lobes. These ERPs recall respectively the N400 elicited by semantic violation and the syntactic positive shift (the P600) argued to index syntactic integration difficulties [44,45] Kaan and Swaab 2003; Osterhout et al. 1994). While all these studies support the existence of syntactic mechanisms at work in complex action understanding, the present study critically adds to our knowledge by indicating that the motor production system could share rather specific structural representations and processes with language as well as, possibly, some of its constraints, namely, perhaps constraints akin to the linguistic Coordinate Structure constraint.

In conclusion, supporting Jackendoff’s theoretical conjecture, we provide experimental evidence for a motor structure that is in many ways analogous to the linguistically characterized distant dependency at hand in relative clauses. Our study is also the first to make use of a task simple enough to be performed by young patients, but whose structure is sufficiently complex to probe fine motor skills. Our task uses a fixed set of material (objects properties and movement goals) and manipulates only one feature of the target action, namely prior knowledge of object weight. Moreover, this task enables a direct access to the structural properties of simple actions without the potential confounds of semantic or cultural factors. Our findings, that a developmental linguistic deficit affecting (among other) the ability to construct complex embeddings and dependencies, is mirrored by a structural deficit in building the motor analogue of a distant dependency strongly restate the principled motivation for investigating common motor and linguistic structural mechanisms, and the existence of a possible motor precursor for language syntax.

## Methods

### Ethics Statement

All participants were naïve as to the purpose of the study and all participants as well as their parents or guardians (for children), gave a written informed consent to participate to the study, which was approved by the local ethics committee (CPP Sud Est II), and were tested in observance of the Declaration of Helsinki.

### Participants

**SLI children** were diagnosed by a trained multidisciplinary team of specialists working at the national reference center for learning and communications disorders of the Lyon hospitals. IQ evaluation revealed a difference of at least 20 points between IQv and IQp (mean score 91.6 and 116.5, respectively), which represents more than 1.5 standard deviations. Patients were between 9 years and 13 years and 4 months, mean age was 11 years. All patients, except one, were right handed. Four patients were diagnosed with a dysphasia affecting the phonological and syntactic aspects of their language, and three patients were diagnosed with a dysphasia affecting the lexical and syntactic aspects. All patients have been undergoing intensive speech reeducation for several years (six years and a half on average). At the time of testing, children had at least partially recovered from phonological and lexical deficits, but remained dramatically impaired at the syntactic level, expressing themselves using simple sentences only. Language production rather than comprehension was affected: For 6 out of the 7 patients syntactic comprehension as assessed with the ECOSSE test (Evaluation de la Compréhension Syntaxico-Sémantique, P. Lecocq, 1996) met the expected age-dependent level of performance. On the production side, 4 out of 7 patients exhibited a delay of, on average, 36.3 months with respect to their chronological age (for syntax and morpho-syntax; TGC-R: Test of Grammatical Closure, Deltour, 1992, 2002). In the remaining 3 patients, the developmental age of syntactic abilities was not quantifiable: Despite a chronological age of 9 years and 1 month, 9 and 13 years, no complex sentences were produced, and the present tense was the only one used.

**FXS patients** were recruited through the Rare Causes of Intellectual Disability National Center of the Lyon hospitals. They were between 20 and 31 years-old. All but one were right-handed. FXS was confirmed by more than 200 CGG repeats or a positive cytogenetic test and a family history of FXS. Mental age varied from 4,5 to 7,5 years as evaluated with nonverbal reasoning test (Raven’s Colored Progressive Matrices). Language comprehension, assessed with the ECOSSE test, revealed a developmental age of 5 years and 5 months. On the production side the mean length of utterance was 5,39 words and the mean age of grammatical development was 4,92 as evaluated with the TGC-R.

**Typically developing children** and **healthy adults** (all right-handed) were recruited out of patient’s relatives.

### Stimuli and procedure

Two visually identical white opaque bottles (250ml containers) were used as stimuli, one weighting 50g (termed hereafter ‘light’) and one weighting 500g (termed hereafter ‘heavy’). Prior to starting the experiment, participants were asked to manually familiarize themselves with the bottle and the experimenter reinforced their perception by saying “You feel how this bottle is heavy/light, now feel this one; isn’t it much lighter/heavier?”.

Participants were required to keep their hand, held in a pinch grip position, on a fixed starting point on a table along their sagittal axis. Upon hearing a go-signal they were instructed to reach and grasp for the bottle (placed 20cm in front of the starting point) and to displace it to a pre-defined position 15cm to the right of the initial position. Once the bottle was displaced to its final position, participants replaced his/her hand back on the starting point and waited for the next go-signal. Participants were instructed to grasp the bottle on its cap to ensure a uniformly sized grasp surface. Participants performed a total of 40 trials. In the first 20 trials, relevant information about object weight (i.e., light vs. heavy) was provided prior to movement onset and participants perform a block of 10 successive trials with the heavy object, and a block of 10 successive trials with the light object, or vice versa. In the remaining 20 trials, object weight was unknown and heavy and light trials were proposed in a pseudo random order. To ensure that participants were oblivious of object weight, experimenter’s manipulation of the bottles was concealed.

### Movement recordings and data processing (analysis)

Movement kinematics were recorded via an Optotrak 3020 system (Northern Digital Inc). One active infrared marker (sampling rate set at 300Hz) was placed on the wrist, two respectively on the index and thumb fingers, and two on the bottles. A second-order Butterworth dual pass filter (cutoff frequency, 10Hz) was used for raw data processing. Individual movements were then visualized and analyzed using Optodisp software (Optodisp copyright UCBL-CNRS, Marc Thevenet et Yves Paulignan, 2001). For each Reach sub-phase latency and amplitude of the first and second acceleration peaks, velocity peaks, deceleration peaks and grip aperture were measured. For the Move sub-phase latency and amplitude of the highest acceleration peaks, velocity peaks, and deceleration peaks were measured (Figure S1). The whole action time, as defined as the time elapsed between the beginning of the movement when participants left the starting point and movement end when participants had displaced the bottle in its final position.

To reduce noise, the first two movements of each of the four experimental conditions were discarded from subsequent analysis. For each participant, mean values for the different kinematic parameter were computed separately for each condition. Data normality and homoscedasticity were controlled with Shapiro-Wilk and Levene tests, respectively. Mean values for each participant were entered into a repeated measures ANOVA with Weight (light, heavy) and Knowledge (known, unknown) as within-subject factors. To further test the combined effect of all measured parameters of the movement a multivariate approach was applied. Since the requirement of a parametric MANOVA, i.e., to have more observations than parameters, is not fulfilled in our case, a resampling-based non parametric MANOVA with Fisher combination of the p-values was used [46,47].

## Supporting Information

Figure S1 **Wrist velocity and acceleration profile for the Displace action task.**
Here are represented the wrist velocity (left panel) and acceleration profile (right panel) pertaining to an individual representative movement and the collected parameters. The Reach sub-phase (green ground) is characteristically composed of two acceleration peaks followed by a velocity peak (red marks) and a deceleration peak (green mark); the ensuing Move Object phase (orange ground) is in turn characterized by an acceleration peak, a velocity peak (red marks) and a deceleration peak (green mark). Please note that more than one deceleration peak may occur for each movement sub-phase (or acceleration for the second sub-phase); in those cases, the lowest deceleration or on the contrary the highest acceleration peak was collected for subsequent analyses.
(TIF)

File S1 **Tables S1 & S2.**
(PPTX)
