# Supplementary figures and images for: Syntax at Hand: Common Syntactic Structures for Actions and Language
Source: PLoS One. 2013 Aug 22;8(8):e72677. doi: 10.1371/journal.pone.0072677 (PMC3749983; doi:10.1371/journal.pone.0072677)

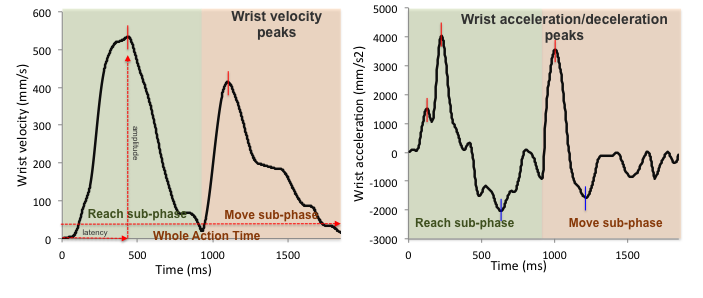

Supplement: Figure S1 — Wrist velocity and acceleration profile for the Displace action task. Here are represented the wrist velocity (left panel) and acceleration profile (right panel) pertaining to an individual representative movement and the collected parameters. The Reach sub-phase (green ground) is characteristically composed of two acceleration peaks followed by a velocity peak (red marks) and a deceleration peak (green mark); the ensuing Move Object phase (orange ground) is in turn characterized by an acceleration peak, a velocity peak (red marks) and a deceleration peak (green mark). Please note that more than one deceleration peak may occur for each movement sub-phase (or acceleration for the second sub-phase); in those cases, the lowest deceleration or on the contrary the highest acceleration peak was collected for subsequent analyses. (TIF) [file pone.0072677.s001.tif]
