# Supplementary material for: Syntax at Hand: Common Syntactic Structures for Actions and Language
Source: PLoS One. 2013 Aug 22;8(8):e72677. doi: 10.1371/journal.pone.0072677 (PMC3749983; doi:10.1371/journal.pone.0072677)
Supplement: File S1 — Tables S1 & S2. (PPTX) [file pone.0072677.s002.pptx]

## Slide 1
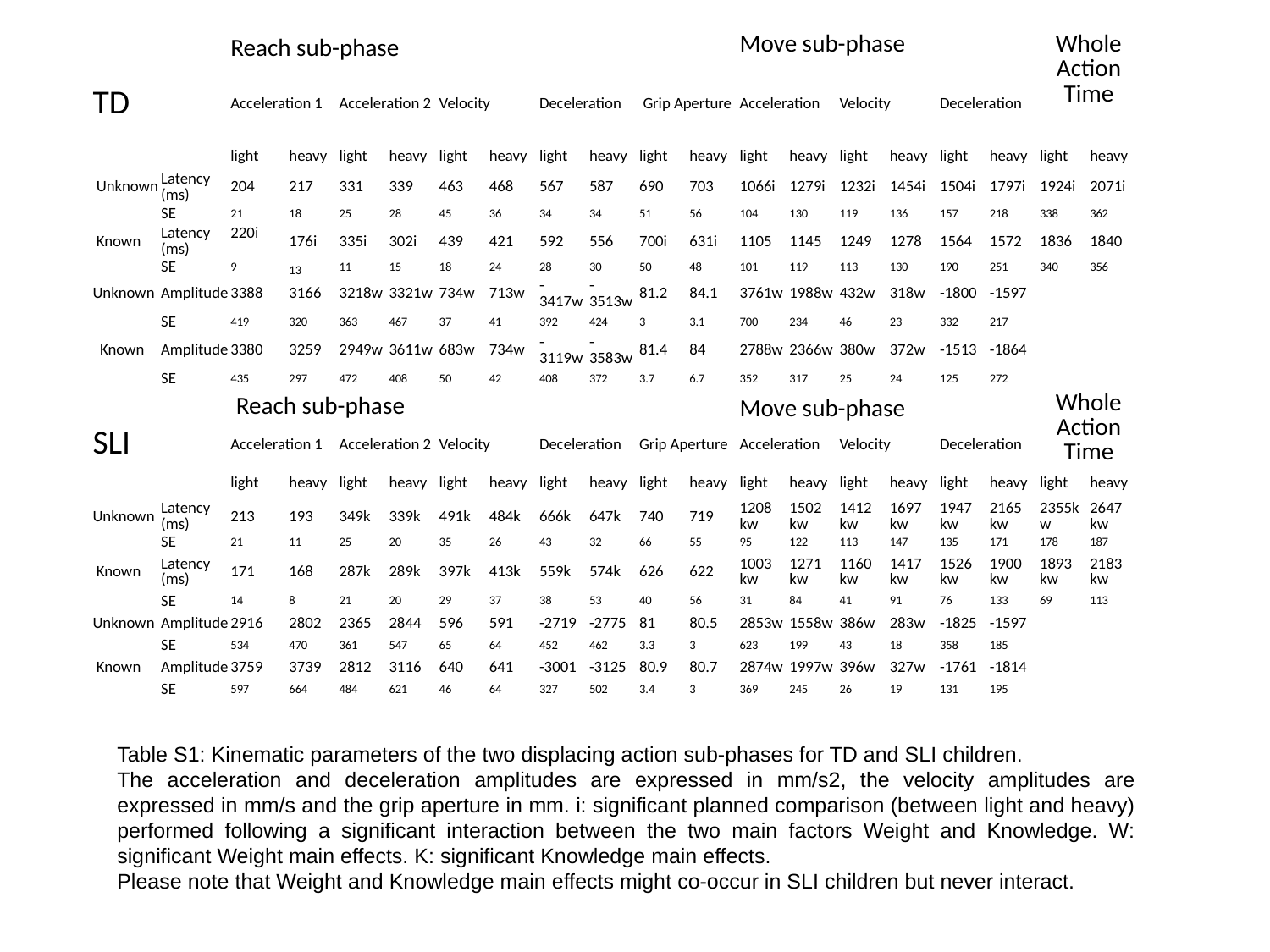

| | | Reach sub-phase | | | | | | | | | | Move sub-phase | | | | | | Whole Action Time | |
| --- | --- | --- | --- | --- | --- | --- | --- | --- | --- | --- | --- | --- | --- | --- | --- | --- | --- | --- | --- |
| TD | | Acceleration 1 | | Acceleration 2 | | Velocity | | Deceleration | | Grip Aperture | | Acceleration | | Velocity | | Deceleration | | | |
| | | light | heavy | light | heavy | light | heavy | light | heavy | light | heavy | light | heavy | light | heavy | light | heavy | light | heavy |
| Unknown | Latency (ms) | 204 | 217 | 331 | 339 | 463 | 468 | 567 | 587 | 690 | 703 | 1066i | 1279i | 1232i | 1454i | 1504i | 1797i | 1924i | 2071i |
| | SE | 21 | 18 | 25 | 28 | 45 | 36 | 34 | 34 | 51 | 56 | 104 | 130 | 119 | 136 | 157 | 218 | 338 | 362 |
| Known | Latency (ms) | 220i | 176i | 335i | 302i | 439 | 421 | 592 | 556 | 700i | 631i | 1105 | 1145 | 1249 | 1278 | 1564 | 1572 | 1836 | 1840 |
| | SE | 9 | 13 | 11 | 15 | 18 | 24 | 28 | 30 | 50 | 48 | 101 | 119 | 113 | 130 | 190 | 251 | 340 | 356 |
| Unknown | Amplitude | 3388 | 3166 | 3218w | 3321w | 734w | 713w | -3417w | -3513w | 81.2 | 84.1 | 3761w | 1988w | 432w | 318w | -1800 | -1597 | | |
| | SE | 419 | 320 | 363 | 467 | 37 | 41 | 392 | 424 | 3 | 3.1 | 700 | 234 | 46 | 23 | 332 | 217 | | |
| Known | Amplitude | 3380 | 3259 | 2949w | 3611w | 683w | 734w | -3119w | -3583w | 81.4 | 84 | 2788w | 2366w | 380w | 372w | -1513 | -1864 | | |
| | SE | 435 | 297 | 472 | 408 | 50 | 42 | 408 | 372 | 3.7 | 6.7 | 352 | 317 | 25 | 24 | 125 | 272 | | |
| | | Reach sub-phase | | | | | | | | | | Move sub-phase | | | | | | Whole Action Time | |
| SLI | | Acceleration 1 | | Acceleration 2 | | Velocity | | Deceleration | | Grip Aperture | | Acceleration | | Velocity | | Deceleration | | | |
| | | light | heavy | light | heavy | light | heavy | light | heavy | light | heavy | light | heavy | light | heavy | light | heavy | light | heavy |
| Unknown | Latency (ms) | 213 | 193 | 349k | 339k | 491k | 484k | 666k | 647k | 740 | 719 | 1208 kw | 1502 kw | 1412 kw | 1697 kw | 1947 kw | 2165 kw | 2355kw | 2647 kw |
| | SE | 21 | 11 | 25 | 20 | 35 | 26 | 43 | 32 | 66 | 55 | 95 | 122 | 113 | 147 | 135 | 171 | 178 | 187 |
| Known | Latency (ms) | 171 | 168 | 287k | 289k | 397k | 413k | 559k | 574k | 626 | 622 | 1003 kw | 1271 kw | 1160 kw | 1417 kw | 1526 kw | 1900 kw | 1893 kw | 2183 kw |
| | SE | 14 | 8 | 21 | 20 | 29 | 37 | 38 | 53 | 40 | 56 | 31 | 84 | 41 | 91 | 76 | 133 | 69 | 113 |
| Unknown | Amplitude | 2916 | 2802 | 2365 | 2844 | 596 | 591 | -2719 | -2775 | 81 | 80.5 | 2853w | 1558w | 386w | 283w | -1825 | -1597 | | |
| | SE | 534 | 470 | 361 | 547 | 65 | 64 | 452 | 462 | 3.3 | 3 | 623 | 199 | 43 | 18 | 358 | 185 | | |
| Known | Amplitude | 3759 | 3739 | 2812 | 3116 | 640 | 641 | -3001 | -3125 | 80.9 | 80.7 | 2874w | 1997w | 396w | 327w | -1761 | -1814 | | |
| | SE | 597 | 664 | 484 | 621 | 46 | 64 | 327 | 502 | 3.4 | 3 | 369 | 245 | 26 | 19 | 131 | 195 | | |
Table S1: Kinematic parameters of the two displacing action sub-phases for TD and SLI children.
The acceleration and deceleration amplitudes are expressed in mm/s2, the velocity amplitudes are expressed in mm/s and the grip aperture in mm. i: significant planned comparison (between light and heavy) performed following a significant interaction between the two main factors Weight and Knowledge. W: significant Weight main effects. K: significant Knowledge main effects.
Please note that Weight and Knowledge main effects might co-occur in SLI children but never interact.

## Slide 2
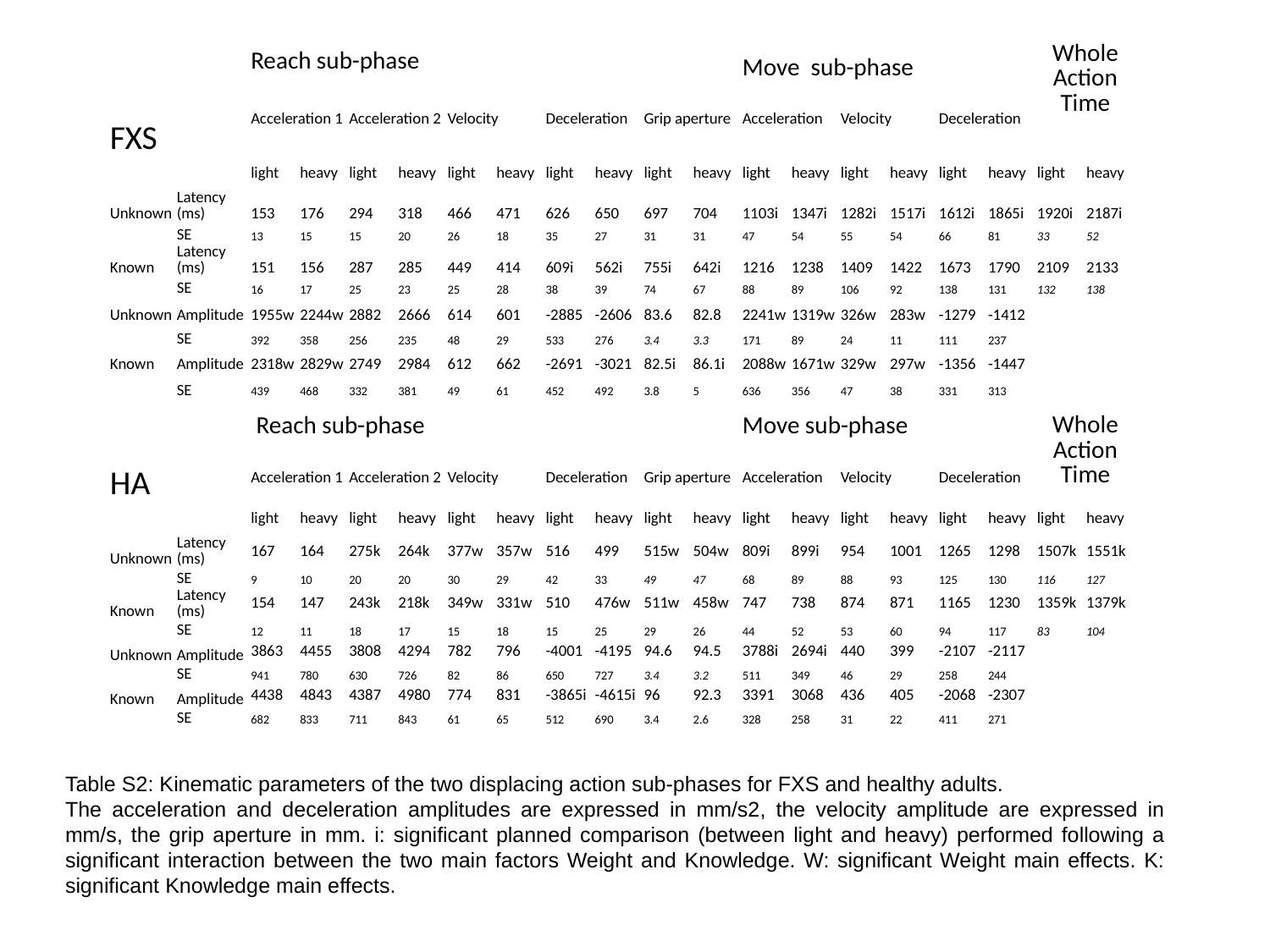

| | | Reach sub-phase | | | | | | | | | | Move  sub-phase | | | | | | Whole Action Time | |
| --- | --- | --- | --- | --- | --- | --- | --- | --- | --- | --- | --- | --- | --- | --- | --- | --- | --- | --- | --- |
| FXS | | Acceleration 1 | | Acceleration 2 | | Velocity | | Deceleration | | Grip aperture | | Acceleration | | Velocity | | Deceleration | | | |
| | | light | heavy | light | heavy | light | heavy | light | heavy | light | heavy | light | heavy | light | heavy | light | heavy | light | heavy |
| Unknown | Latency (ms) | 153 | 176 | 294 | 318 | 466 | 471 | 626 | 650 | 697 | 704 | 1103i | 1347i | 1282i | 1517i | 1612i | 1865i | 1920i | 2187i |
| | SE | 13 | 15 | 15 | 20 | 26 | 18 | 35 | 27 | 31 | 31 | 47 | 54 | 55 | 54 | 66 | 81 | 33 | 52 |
| Known | Latency (ms) | 151 | 156 | 287 | 285 | 449 | 414 | 609i | 562i | 755i | 642i | 1216 | 1238 | 1409 | 1422 | 1673 | 1790 | 2109 | 2133 |
| | SE | 16 | 17 | 25 | 23 | 25 | 28 | 38 | 39 | 74 | 67 | 88 | 89 | 106 | 92 | 138 | 131 | 132 | 138 |
| Unknown | Amplitude | 1955w | 2244w | 2882 | 2666 | 614 | 601 | -2885 | -2606 | 83.6 | 82.8 | 2241w | 1319w | 326w | 283w | -1279 | -1412 | | |
| | SE | 392 | 358 | 256 | 235 | 48 | 29 | 533 | 276 | 3.4 | 3.3 | 171 | 89 | 24 | 11 | 111 | 237 | | |
| Known | Amplitude | 2318w | 2829w | 2749 | 2984 | 612 | 662 | -2691 | -3021 | 82.5i | 86.1i | 2088w | 1671w | 329w | 297w | -1356 | -1447 | | |
| | SE | 439 | 468 | 332 | 381 | 49 | 61 | 452 | 492 | 3.8 | 5 | 636 | 356 | 47 | 38 | 331 | 313 | | |
| | | Reach sub-phase | | | | | | | | | | Move sub-phase | | | | | | Whole Action Time | |
| HA | | Acceleration 1 | | Acceleration 2 | | Velocity | | Deceleration | | Grip aperture | | Acceleration | | Velocity | | Deceleration | | | |
| | | light | heavy | light | heavy | light | heavy | light | heavy | light | heavy | light | heavy | light | heavy | light | heavy | light | heavy |
| Unknown | Latency (ms) | 167 | 164 | 275k | 264k | 377w | 357w | 516 | 499 | 515w | 504w | 809i | 899i | 954 | 1001 | 1265 | 1298 | 1507k | 1551k |
| | SE | 9 | 10 | 20 | 20 | 30 | 29 | 42 | 33 | 49 | 47 | 68 | 89 | 88 | 93 | 125 | 130 | 116 | 127 |
| Known | Latency (ms) | 154 | 147 | 243k | 218k | 349w | 331w | 510 | 476w | 511w | 458w | 747 | 738 | 874 | 871 | 1165 | 1230 | 1359k | 1379k |
| | SE | 12 | 11 | 18 | 17 | 15 | 18 | 15 | 25 | 29 | 26 | 44 | 52 | 53 | 60 | 94 | 117 | 83 | 104 |
| Unknown | Amplitude | 3863 | 4455 | 3808 | 4294 | 782 | 796 | -4001 | -4195 | 94.6 | 94.5 | 3788i | 2694i | 440 | 399 | -2107 | -2117 | | |
| | SE | 941 | 780 | 630 | 726 | 82 | 86 | 650 | 727 | 3.4 | 3.2 | 511 | 349 | 46 | 29 | 258 | 244 | | |
| Known | Amplitude | 4438 | 4843 | 4387 | 4980 | 774 | 831 | -3865i | -4615i | 96 | 92.3 | 3391 | 3068 | 436 | 405 | -2068 | -2307 | | |
| | SE | 682 | 833 | 711 | 843 | 61 | 65 | 512 | 690 | 3.4 | 2.6 | 328 | 258 | 31 | 22 | 411 | 271 | | |
Table S2: Kinematic parameters of the two displacing action sub-phases for FXS and healthy adults.
The acceleration and deceleration amplitudes are expressed in mm/s2, the velocity amplitude are expressed in mm/s, the grip aperture in mm. i: significant planned comparison (between light and heavy) performed following a significant interaction between the two main factors Weight and Knowledge. W: significant Weight main effects. K: significant Knowledge main effects.
